# Bibliometric description of the literature on HTLV-1 registered in PubMed over the past ten years

**DOI:** 10.1186/1742-4690-8-S1-A247

**Published:** 2011-06-06

**Authors:** Kristien Verdonck, Eduardo Gotuzzo

**Affiliations:** 1 Instituto de Medicina Tropical Alexander von Humboldt, Universidad Peruana Cayetano Heredia, Lima, Peru; 2 Institute of Tropical Medicine, Antwerp, Belgium

## Introduction

We conducted a bibliometric evaluation to get insight into research activities and tendencies.

## Methods

The search criteria in PubMed were: (1) “human T-lymphotropic virus 1” as medical subject headings (MeSH) major topic; and (2) publication date from January 2001-December 2010. We retrieved publication year, author names, journal, country of first author, language, free availability, and MeSH terms.

## Results

The search retrieved 1189 publications by 3969 authors. Twenty-eight authors published >=15 articles. The number of publications did not increase: 600 in 2001-2005 and 589 in 2006-2010. The journals with the highest number of publications are: Journal of virology (126), Retrovirology (58), Blood (47), The Journal of biological chemistry (45) and Virology (41). In 349 publications (30%), the first author was from the USA; Japan contributed 329 publications (28%); Brazil 115 (10%); France 91 (8%); and the UK 54 (5%). The proportion of publications with a first author from Latin America or the Caribbean increased over time (10% in 2001-2002; 18% in 2009-2010); the proportion with a North American first author decreased (34% in 2001-2002; 26% in 2009-2010). Ninety-six percent (1137) of the publications are in English. The full text of 582 publications (49%) is freely available. The most frequent MeSH terms relate to virology. Clinical/epidemiological MeSH terms are more frequent in publications from Latin America and the Caribbean than elsewhere.

## Conclusions

The constantly modest number of publications characterizes HTLV-1 as a neglected health problem. The MeSH terms suggest that clinical/epidemiological aspects are investigated much less frequently than virological aspects.

